# Cardiovascular event-free survival after adjuvant radiation therapy in breast cancer patients stratified by cardiovascular risk

**DOI:** 10.1002/cam4.283

**Published:** 2014-07-10

**Authors:** Nneka C Onwudiwe, Young Kwok, Eberechukwu Onukwugha, John D Sorkin, Ilene H Zuckerman, Fadia T Shaya, C Daniel Mullins

**Affiliations:** 1 Department of Pharmaceutical Health Services Research, University of Maryland School of PharmacyBaltimore, Maryland; 2 Department of Radiation Oncology, University of Maryland School of MedicineBaltimore, Maryland; 3 Geriatrics Research, Education and Clinical Center, Baltimore VA Medical CenterBaltimore, Maryland; 4 Division of Gerontology and Geriatric Medicine, Department of Medicine, University of Maryland School of MedicineBaltimore, Maryland

**Keywords:** Breast cancer, cardiotoxicity, cardiovascular risk, radiation therapy, survival

## Abstract

The objective of this study was to estimate the risk of a cardiovascular event or death associated with modern radiation in a population of elderly female breast cancer patients with varying baseline cardiovascular risk. The data used for this analysis are from the linked Surveillance, Epidemiology, and End-Results (SEER)-Medicare database. The retrospective cohort study included women aged 66 years and older with stage 0–III breast cancer diagnosed between 2000 and 2005. Women were grouped as low, intermediate, or high cardiovascular risk based on the presence of certain clinical diagnoses. The risk for the combined outcome of a hospitalization for a cardiovascular event or death within 6 months and 24 months of diagnosis was estimated using a multivariable Cox model. The median follow-up time was 24 months. Among the 91,612 women with American Joint Committee on Cancer (AJCC) stage 0–III breast cancer: 39,555 (43.2%) were treated with radiation therapy and 52,057 (56.8%) were not. The receipt of radiation therapy in the first 6 months was associated with a statistically significant increased risk for the combined outcome in women categorized as high risk (HR = 1.510; 95% CI, 1.396–1.634) or intermediate risk (HR = 1.415; 95% CI, 1.188–1.686) but not low risk (HR = 1.027; 95% CI, 0.798–1.321). Women with a prior medical history of cardiovascular disease treated with radiation therapy are at increased risk for an event and should be monitored for at least 6 months following treatment with radiation therapy.

## Introduction

Mortality from breast cancer in the United States has declined by an estimated 25–38% in part because of screening and adjuvant treatment 1. In 2009, there were ∼2.8 million breast cancer survivors in the United States (women diagnosed with cancer of the breast at any point prior to 1 January, 2010) 2. Many older women diagnosed with early-stage breast cancer and who survive the breast cancer have a risk of death due to cardiovascular disease 3. There is mounting evidence of a relation between breast cancer treatments and the risk of late toxicities, including cardiovascular events 4–6.

Treatment for breast cancer consists of a combination of surgical mastectomy or lumpectomy, axillary lymph-node dissection, radiotherapy, and systemic hormone therapy or chemotherapy 7. Radiation therapy, also called radiotherapy, in breast cancer treatment uses ionizing radiation to kill malignant cells 8. Treatment with tangential breast irradiation after breast conservation surgery (BCS) has proven to be an effective treatment option in women with breast cancer 9. Several studies have shown that radiotherapy as a treatment option in breast cancer reduces the local-regional recurrence rate 10–12 and improves survival 13,14. Yet, the proven beneficial effects of radiotherapy in reducing breast cancer death and recurrence are offset by increased risk of cardiovascular disease. Empirical evidence documents an increased risk of cardiovascular morbidity and mortality 5,15–21 in breast cancer patients, especially among patients with left-side breast cancer 5,20,22. Although early epidemiologic studies have reported increased cardiovascular morbidity and mortality related to the use of left-sided irradiation, it must be recognized that right-side breast irradiation does expose the heart to radiation 23, and so there is a risk for cardiotoxicity related to the use of right-sided irradiation. Acute pericarditis, pericardial effusion, and arrhythmias are the most frequently diagnosed cardiotoxicity of adjuvant radiotherapy 24.

Despite advances in radiation treatment for breast cancer which limit radiation exposure, for many women with breast cancer, exposing the heart to radiation will be unavoidable. Cardiovascular complications from cancer therapy can occur immediately, or manifest months or years after the patient has been treated 25. Estimating the risk of an event, and stratifying patients according to cardiovascular risk for these events would be useful in identifying those patients most likely to benefit from management plans, as well as strategies to reduce cardiotoxicity. The goal of this study was to expand upon prior research estimating the effect of adjuvant radiation therapy on cardiovascular event-free survival (EFS) by stratifying patients according to baseline cardiovascular risk.

## Patients and Methods

### Data source

The data used for this analysis are from the linked Surveillance, Epidemiology, and End-Results (SEER)-Medicare database. The database links records from SEER, a population-based cancer registry, and Medicare, the federally funded health insurance program for elderly persons age 65 and over. The SEER registry covers ∼28% of the US population and is comparable to the general US population with regard to measures of poverty and education. SEER tracks all incident cancer cases and survival in the US among residents in selected geographic areas. The SEER registry collects information on patient demographics, primary tumor site, tumor morphology, and stage at diagnosis, along with cancer-directed surgery and radiation therapy provided for first course of treatment (http://seer.cancer.gov) 26.

The Medicare claims database provides a complete expenditure and source of payment data on all health care services for beneficiaries 65 years or older, under age 65 with certain disabilities, and people of all ages with end-stage renal disease (ESRD). The linkage of the two data sources provides a unique source of clinical, demographic, and cause of death information for persons with cancer and claims for covered health care services.

### Patient selection criteria

We conducted a retrospective analysis of women, age 66 years and older with American Joint Committee on Cancer (AJCC) stage 0–III breast cancer diagnosed between 2000 and 2005. The main eligibility criteria were continuous participation in Medicare Parts A and B during any month of the study period; age ≥66 years to allow for a 1-year time period following Medicare enrollment during which comorbidities could be assessed; AJCC stage 0–III breast cancer; and treatment with surgical therapy with or without adjuvant radiotherapy within 52 weeks after diagnosis. We excluded the following: men; women enrolled in a health maintenance organization (HMO) because data were unavailable for these periods; women whose Medicare eligibility was on the basis of disability (DIB) or ESRD; women with bilateral disease or unknown laterality; AJCC stage IV or unstaged disease; women with a prior cancer diagnosis; and women in whom other cancers were diagnosed within 12 months of breast cancer diagnosis. For each patient, we utilized claims information available from 12 months prior to breast cancer diagnosis to date of death or censoring on 31 December, 2007.

### Measurement of treatments, outcomes, and covariates

The primary outcome in this study was cardiovascular EFS. Events were hospitalization for a cardiovascular event and death. Mortality in this study was defined as death from any cause. Hospitalization for a cardiovascular event was determined using International Classification of Disease, ninth revision, Clinical Modification (ICD-9-CM) codes (Data S1) in the first, second, or third position for an inpatient hospital or skilled nursing facility (SNF) claim. Hospitalization for a cardiovascular event was ascertained using ICD-9-CM codes in the Medicare Provider Analysis and Review (MEDPAR) file. Ascertainment of cardiovascular diagnoses within 12 months before the diagnosis of breast cancer was used to define the baseline cardiovascular risk category; cardiovascular diagnoses identified after the breast cancer diagnosis were considered to be radiation-associated cardiovascular events. For this analysis, a cardiovascular event was defined as a diagnosis of angina, heart failure, chest pain, ischemia, valve disorder, heart disease, atherosclerosis, cardiac inflammation, conduction/rhythm disorder, myocardial infarction (MI), cardiomyopathy, or elevated blood pressure.

We categorized women into three mutually exclusive groups according to their baseline risk for a cardiovascular event: high risk, intermediate risk, and low risk. A clinical diagnosis of cardiovascular disease or its risk factors was used to group women into these risk categories. Women with clinical cardiovascular disease fell into the high-risk category. Women with one or multiple risk factors and no diagnosis of cardiovascular disease fell into the intermediate risk category. A cardiovascular risk factor was defined as having an ICD-9-CM diagnosis code for tobacco dependence, diabetes, hypercholesterolemia, obesity, chronic kidney disease (CKD), and/or hypertension. Women not meeting the criteria for high or intermediate risk were categorized as low risk.

Radiotherapy exposure was defined as whole breast irradiation (WBI, or tangential field external beam radiation). Documentation of radiotherapy treatment in the SEER file and Medicare claims file was used to identify women receiving radiation therapy. Because of the risk of discordance on radiation therapy information between the SEER and Medicare claims, women were identified as having received whole breast radiotherapy if they had the SEER codes for beam radiation or combination of beam with implants or isotopes, but not if they only had radioactive implants, radioisotopes, or other radiation method. In addition to SEER fields of radiation treatment within 4 months of diagnosis, women whose treatment did not fall within the 4-month window were further identified as having successfully received whole breast radiotherapy if they had a Medicare ICD-9-CM, Healthcare Common Procedure Coding System (HCPCS) or Current Procedural Terminology (CPT) code for radiation. Women with at least one radiotherapy code within 12 months from the date of diagnosis were identified as having received whole breast radiotherapy.

Partial mastectomy or BCS exposure included nipple resection, lumpectomy or excisional biopsy, re-excision of biopsy site, wedge resection, quadrantectomy, segmental mastectomy, or tylectomy. Mastectomy exposure included subcutaneous, total, modified radical mastectomy with-or-without removal of uninvolved contralateral breast, radical mastectomy with-or-without removal of uninvolved contralateral breast, extended radical mastectomy with-or-without removal of uninvolved contralateral breast, or mastectomy not otherwise specified (NOS). Women were considered to have undergone surgery if a claim was recorded in Medicare, but if a patient had only a SEER code for surgery and not a Medicare claim for surgery the patient was then categorized as receiving surgery if they had a SEER code.

Medicare claims were the source of chemotherapy exposure. Chemotherapy exposure was ascertained using ICD-9-CM V codes (V581 V672 V662), CPT (964XX, 965XX), and HCPCS codes (Q0083-Q0085, J9000-J9999, G09XX, C89XX-C94XX).

Covariates such as age, marital status, geographic region, year of diagnosis, laterality, AJCC staging, and race were obtained from SEER records. The date of cancer diagnosis was combined with the Medicare date of birth, presented as month and year (but not day, to protect privacy) in the Patient Entitlement and Diagnosis Summary File (PEDSF) file, to compute age at cancer diagnosis. The identification of racial groups in this study used the recoded SEER race variable, which combines information from race and Hispanic surname variables. Because tumor characteristics grouped into the SEER AJCC designated staging variable by TNM classification is based on the 3rd edition of the AJCC manual for cases diagnosed between 1988 and 2003 and the derived AJCC 6th edition for cases diagnosed 2004 and beyond, the two SEER AJCC staging variables were combined to compute a staging variable for analysis.

The Charlson comorbidity index adapted by Deyo 27 was used to calculate a comorbidity index for each patient with respect to cancer. Claim records from the date of diagnosis minus 12 months through the date of diagnosis minus 1 month were used to compute the comorbidity index.

### Statistical analysis

The chi-square test was used to compare frequency distributions of baseline and clinical characteristics of categorical variables within the entire population. Using the combined endpoint of death and cardiovascular event, cardiovascular EFS curves were generated for each group using the Kaplan–Meier survival function. For each group of women, the log-rank test was used to compare cardiovascular EFS. Cardiovascular EFS in this study was defined as the proportion of women who remain alive and free of a cardiovascular event necessitating hospital admission in the first 6 months and 24 months following diagnosis.

Multivariable Cox models were used to estimate the effect of radiation on cardiovascular EFS. The regression models included an interaction variable between radiation and time to investigate nonproportional hazards associated with radiation receipt. Covariate selection was based on clinical importance in prior studies. Separate models were created for all three cardiovascular risk groups. Estimation of hazard ratios with 95% CIs was obtained using maximum partial likelihood estimation. All statistical analyses were performed using SAS version 9.3 (SAS Institute, Cary, NC). Alpha was set at *P* < 0.05 to define statistical significance.

## Results

The SEER-Medicare database included a total of 177,092 breast cancer cases from 2000 to 2005. We excluded the following: 1382 men; 53,446 women age <66 years at breast cancer diagnosis; 398 women whose Medicare eligibility was based on DIB or ESRD; 15,643 women with stage IV breast cancer; 10,777 women with a prior cancer diagnosis; 1845 women in whom other cancers were diagnosed within 12 months of breast cancer diagnosis; 40 women with bilateral disease or unknown laterality; 747 women enrolled in an HMO; and 1202 women who received partial breast radiation or radiation NOS.

The final study cohort consisted of 91,612 women with AJCC stage 0-III breast cancer diagnosed between 2000 and 2005. Of the 91,612 women included in this study, 52,057 (56.8%) did not receive radiation treatment and 39,555 (43.2%) received radiation treatment. The median time from breast cancer diagnosis to start of radiotherapy was 2 months. The median follow-up time was 24 months. The mean age at diagnosis was 76 years for women with no radiation therapy and 75 years in the radiation therapy group. Women treated with radiotherapy were more likely to receive treatment with chemotherapy (32.2% vs. 11.2%, *P* ≤ 0.0001) compared to women who did not receive radiotherapy (Table 1). Greater proportions of women with cardiovascular disease risk factors and prior cardiovascular disease were found in the radiation-treated group compared to women who did not receive radiation. Women who underwent partial mastectomy were commonly treated with local adjuvant radiotherapy treatment compared to women who underwent mastectomy (78.6% vs. 44.2%, *P* ≤ 0.0001). The percentage of women receiving radiation was lower at higher levels of comorbidity. Women with more advanced age were less likely to receive radiation compared to younger women. There was a lower percentage of women receiving radiation across all race–ethnicity groups compared to whites. At diagnosis, a greater percentage of women with stage I breast cancer received radiation compared to women diagnosed at a higher disease stage.

**Table 1:** Treatment characteristics by radiation therapy status among female breast cancer patients aged 66 years and older, SEER-Medicare data from 1999–2007 (91,612)

	No radiation therapy		Radiation therapy		
			
	N	(%)	N	(%)	*P*-values^1^
Entire cohort	52,057	56.8	39,555	43.2	
Mean age (SD)	76 (7)		75 (6)		
Chemotherapy					
No	46,218	88.8	26,833	67.8	<0.0001
Yes	5839	11.2	12,722	32.2	
Preexisting cardiovascular disease					
No	36,060	69.3	19,602	49.6	<0.0001
Yes	15,997	30.7	19,953	50.4	
Preexisting cardiovascular disease risk factors^2^					
No	30,265	58.1	9267	23.4	<0.0001
Yes	21,792	41.9	30,288	76.6	
Laterality					
Right	25,320	48.6	19,277	48.7	0.7741
Left	26,737	51.4	20,278	51.3	
AJCC Stage					
0	10,237	19.7	5950	15.0	<0.0001
I	22,679	43.6	19,096	48.3	
II	16,114	31.0	11,329	28.6	
III	3027	5.8	3180	8.0	
Surgery					
No Surgery of 1 Site	1698	3.3	653	1.7	<0.0001
Partial Mastectomy/Lumpectomy	23,031	44.2	31,080	78.6	
Mastectomy	27,244	52.3	7777	19.7	
Surgery NOS	84	0.2	45	0.1	
Charlson Comorbidity Score					
Unknown	23,837	45.8	2345	5.9	
0	18,256	35.1	25,956	65.6	
1	6393	12.3	7837	19.8	
2	2234	4.3	2310	5.8	
3+	1337	2.6	1107	2.8	
Year of diagnosis					
2000	9267	17.8	6561	16.6	<0.0001
2001	9374	18.0	6963	17.6	
2002	8984	17.3	6872	17.4	
2003	8042	15.5	6570	16.6	
2004	8043	15.5	6433	16.3	
2005	8347	16.0	6156	15.6	
Age at diagnosis					
66–74	23,125	44.4	21,048	53.2	<0.0001
75–84	21,446	41.2	16,026	40.5	
85+	7486	14.4	2481	6.3	
Race					
White	42,179	81.0	33,561	84.9	<0.0001
Black	3594	6.9	2568	6.5	
Hispanic	3043	5.9	1706	4.3	
Other	3241	6.2	1720	4.4	
Marital Status					
Unmarried	30,239	58.1	20,562	52.0	<0.0001
Married	21,818	41.9	18,993	48.0	
Region					
Midwest	4857	9.3	5427	13.7	<0.0001
West	32,176	61.8	18,517	46.8	
Northeast	7504	14.4	9660	24.4	
South	7520	14.5	5951	15.0	

AJCC, American Joint Committee on Cancer; NOS, Not otherwise specified.

1 Chi-Square Test.

2 Risk Factors: Tobaco dependence, Diabetes, Hyperlipidemia, Hypertension, Obesity, Chronic Kidney Disease.

**Figure 1:**
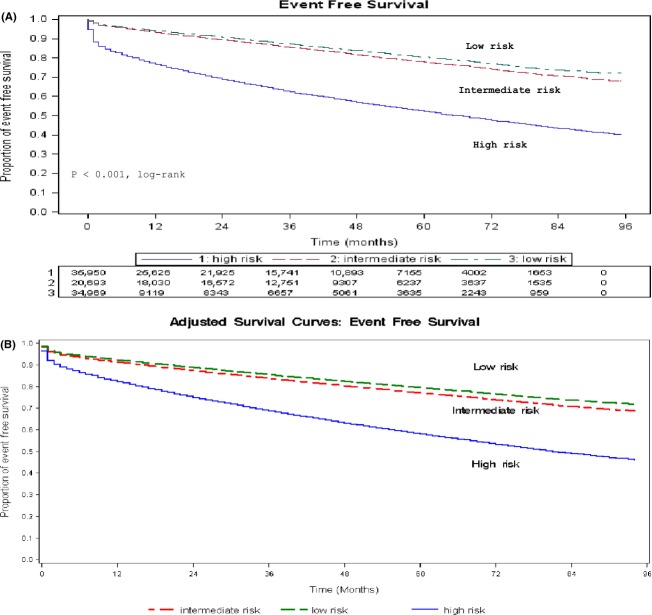
(A) Kaplan–Meier event-free survival estimates over time for the 91,612 women stratified by cardiovascular risk at baseline. (B) Event-free survival estimates over time for the 91,612 women stratified by cardiovascular risk at baseline.

Unadjusted EFS curves were generated using Kaplan–Meier analysis for three groups of women (Fig.1A). Overall, the difference in EFS among the low-risk, intermediate-risk, and high-risk groups was statistically significant by log-rank test (*P* < 0.0001). By pairwise comparison**,** women in the low-risk group were event free longer than those in the intermediate-risk group (*P* < 0.0001), who in turn fared better than those in the high-risk group (*P* < 0.0001). To account for unbalanced distribution of confounders, adjusted comparison of the survival experience of the three risk groups was generated (Fig.1B).

**Figure 2:**
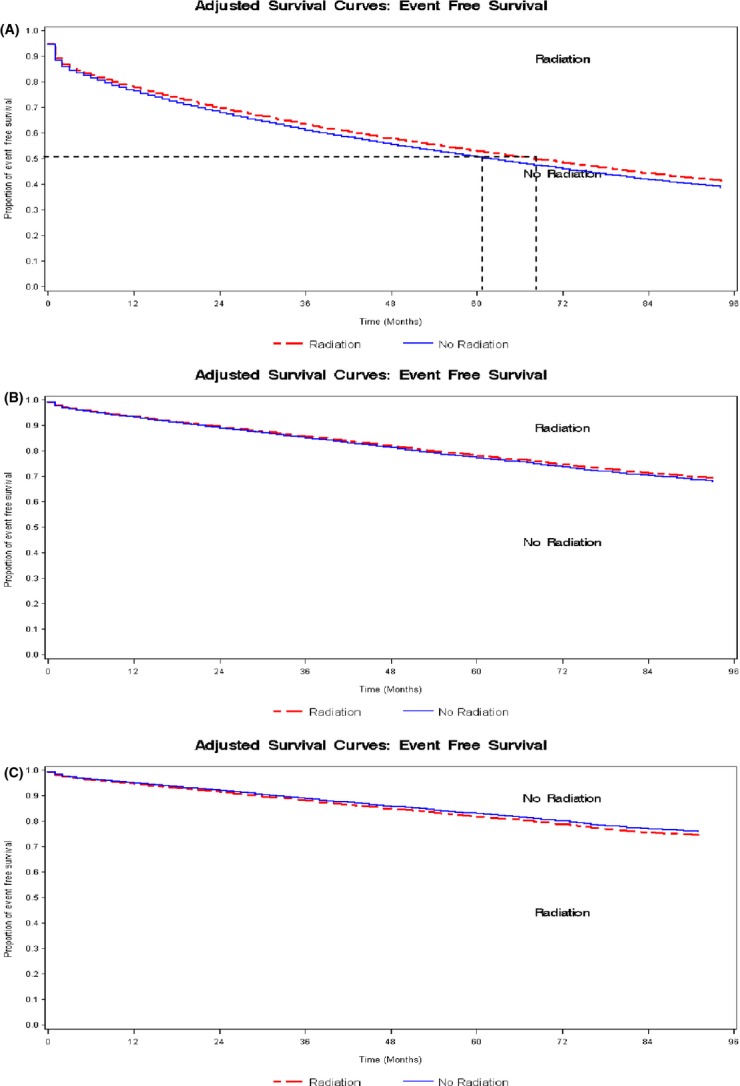
(A) Event-free survival estimates over time for the 35,950 at high risk stratified by radiation treatment. (B) Event-free survival estimates over time for the 20,693 at intermediate risk stratified by radiation treatment. (C) Event-free survival estimates over time for the 34,969 at low risk stratified by radiation treatment.

Figure2 shows adjusted EFS curves comparing two treatment groups—radiotherapy-treated women and no radiotherapy-treated women—within each cardiovascular risk group. Among 35,950 women categorized as high risk, the EFS was longer for women treated with radiotherapy compared with no radiotherapy. The median EFS time for the high-risk group is shown and represents the time at which *S*(*t*) is 0.5. The radiotherapy-treated group had a median EFS time of 68 months (5.7 years), as opposed to 61 months (5.1 years), in the no radiotherapy-treated group, providing some evidence of survival benefit with radiation treatment. The median event-free survival time for women in the intermediate and low-risk group was longer than 96 months (8 years) and difference in EFS between treatment groups was small.

Table 2 shows the proportion of women hospitalized for a particular type of cardiovascular event by risk group. The incidence of events was highest in women in the high-risk group compared to any other cardiovascular risk group.

**Table 2:** Cardiovascular events by baseline cardiovascular risk among female breast cancer patients

	% of patient		
	
	High risk (*n* = 35,950)	Intermediate risk (*n* = 34,969)	Low risk (*n* = 20,693)
Angina	75.28	12.53	12.19
Dyspnea	59.62	17.31	23.08
Heart failure	70.22	15.84	13.94
Chest pain	60.77	21.59	17.64
Ischemia	74.7	14.11	11.19
Valve disorder, nonrheumatic	67.24	16.64	16.12
Atherosclerosis	56.95	24.07	18.97
Cardiac inflammation^1^	53.97	20.63	25.4
Conduction/rhythm disorder	69.22	15.8	14.98
Myocardial infarction	62.59	20.95	16.46
Cardiomyopathy and elevated blood pressure^2^	69.78	13.86	16.36
All-cause mortality	48.55	16.54	34.91

Summary of patients hospitalized for cardiovascular events using ICD-9-CM coding algorithms and proportion deaths due to any cause.

1 Inflammation: Endocarditis, Myocarditis, Pericarditis.

2 Elevated blood pressure with no diagnosis of hypertension.

In the multivariable analysis (Table 3), the risk for the combined outcome (cardiovascular event or death) in the first 6 months and in the first 24 months was estimated. Using radiation as a time-varying predictor, the risk for the combined outcome was higher in the earlier time period than in the later time period, that is, the effect of radiation on the risk for the combined outcome appears to attenuate in the later time period. From Table 3, the effect of radiation in the first 6 months significantly increases the risk (HR = 1.510; 95% CI, 1.396–1.634) for the combined outcome in high-risk women. The estimated hazard ratios, based on the model in Table 3, for the effect of radiation shows a 51% increased risk for the combined outcome in the period up to 6 months of follow-up in high-risk women and no increased risk in the period after 6 months of follow-up. For intermediate risk women, the estimated hazard ratio in the period up to 6 months of follow-up shows a 42% risk for the combined outcome and no increased risk in the period after 6 months of follow-up. The effect of radiation in the period up to 24 months of follow-up showed no increased risk in all three cardiovascular risk groups. In low-risk women, the estimated hazard ratios did not show an increased risk for the combined outcome in either period.

**Table 3:** Multivariate Cox regression model nonproportional hazards using time by radiation interaction for association between risk factors and the combined endpoint of death or cardiovascular disease stratified by baseline cardiovascular risk (91,612)

	Hazard ratio (95% CI)		
	
Cardiac events parameter	High risk (*n* = 15,116)	Intermediate risk (*n* = 3954)	Low risk (*n* = 1956)
Radiotherapy			
No	1	1	1
Yes	0.723 (0.685–0.763)	0.746 (0.650–0.857)	1.007 (0.838–1.211)
Radiotherapy interaction^1^			
Radiotherapy × Time_6_ months	1.510 (1.396–1.634)	1.415 (1.188–1.686)	1.027 (0.798–1.321)
Radiotherapy × Time_24_ months	1.035 (0.950–1.127)	0.962 (0.827–1.118)	1.099 (0.879–1.375)
Chemotherapy			
No	1	1	1
Yes	1.104 (1.062–1.148)	1.252 (1.160–1.351)	1.282 (1.148–1.431)
Laterality			
Right	1	1	1
Left	1.039 (1.006–1.073)	1.064 (1.000–1.133)	0.943 (0.863–1.031)
AJCC stage			
0	1	1	1
I	1.168 (1.112–1.227)	1.101 (1.005–1.207)	1.109 (0.957–1.285)
II	1.261 (1.196–1.330)	1.192 (1.075–1.322)	1.206 (1.031–1.411)
III	1.293 (1.194–1.399)	1.753 (1.501–2.048)	1.654 (1.359–2.013)
Surgery			
No surgery of 1° site	1	1	1
Partial mastectomy/lumpectomy	0.839 (0.759–0.927)	0.860 (0.684–1.082)	0.626 (0.493–0.793)
Mastectomy	1.173 (1.063–1.294)	1.004 (0.800–1.260)	0.793 (0.632–0.994)
Surgery NOS	0.991 (0.671–1.463)	0.750 (0.237–2.378)	0.892 (0.389–2.045)
Charlson comorbidity score			
Unknown/zero^2^	1	1	1
1^1^	1.495 (1.439–1.553)	1.456 (1.352–1.568)	1.383 (1.174–1.631)
2	2.121 (2.020–2.227)	1.911 (1.641–2.225)	1.926 (1.263–2.936)
3^1^	3.130 (2.959–3.311)	3.159 (2.271–4.395)	1.591 (0.512–4.948)
Year of diagnosis			
2000	1.172 (1.103–1.245)	1.315 (1.153–1.500)	1.345 (1.104–1.639)
2001	1.133 (1.067–1.203)	1.280 (1.125–1.457)	1.225 (1.004–1.495)
2002	1.131 (1.065–1.202)	1.181 (1.036–1.346)	1.348 (1.102–1.648)
2003	1.089 (1.023–1.159)	1.120 (0.979–1.282)	1.142 (0.923–1.413)
2004	1.055 (0.990–1.124)	1.040 (0.905–1.195)	1.032 (0.824–1.292)
2005	1	1	1
Age at diagnosis			
66–74	1	1	1
75–84	1.443 (1.390–1.497)	1.585 (1.480–1.698)	1.659 (1.501–1.834)
>85	1.992 (1.894–2.096)	2.485 (2.229–2.771)	3.255 (2.821–3.756)
Race			
White	1	1	1
Black	0.904 (0.850–0.961)	1.106 (0.981–1.246)	1.071 (0.875–1.311)
Hispanic	0.813 (0.742–0.890)	0.968 (0.815–1.150)	1.102 (0.866–1.402)
Other	0.677 (0.610–0.750)	0.742 (0.621–0.887)	0.680 (0.518–0.892)
Marital status			
Unmarried	1	1	1
Married	0.916 (0.884–0.948)	0.823 (0.771–0.879)	0.868 (0.790–0.955)
Region			
Midwest	1	1	1
West	0.864 (0.824–0.907)	0.925 (0.841–1.017)	0.823 (0.717–0.945)
Northeast	0.953 (0.905–1.004)	1.034 (0.931–1.147)	1.053 (0.902–1.230)
South	1.028 (0.974–1.085)	1.080 (0.973–1.199)	1.099 (0.941–1.285)

AJCC, American Joint Committee on Cancer; NOS, Not otherwise specified; CI, Confidence Interval. Likelihood ratio (LR) test (RAD X TIME): 106.06, *P* < 0.0001. High risk: BC patients with CVD disease, Intermediate risk: BC patients with CVD risk factors, Low risk: BC patients with no disease and no risk factors

1 Effect in the first 6 months (RT ^*^ Time), Effect in the first 24 months (RT ^*^ Time), Median follow-up time = 24 months.

2 CCI Category includes those who have a missing CCI and zero CCI.

Other independent predictors for the combined outcome are shown in Table 3. An increased risk was associated with chemotherapy use, earlier calendar year of cancer diagnosis, older age at cancer diagnosis, left-sided tumors, higher AJCC stage, and a higher number of chronic comorbid conditions. Being married was associated with a decreased risk for the combined outcome.

## Discussion

This study found an association between radiation therapy and the combined outcome of death or cardiovascular disease. The analysis showed that elderly women with breast cancer who had preexisting cardiovascular disease and receiving radiation treatment are at a greater risk for the combined outcome in the first 6 months. While there has been little debate regarding radiotherapy-related risk of cardiovascular disease associated with older regimens, results involving more modern regimens are inconclusive. In many studies of radiotherapy-related risk of cardiovascular disease, the risk has been compared between left-sided and right-sided tumors 5,15,20,21,28–30, but this is among the first study to assess the differential risk among older women on the basis of high, intermediate, and low baseline cardiovascular risk. This study found that modern radiation therapy poses greater increases in risk for the combined outcome, especially among women with a significant history of cardiovascular disease. While there are subsets of women who may still be at an increased risk for radiation-induced cardiovascular events, several studies have shown that modern radiation has reduced the risk of cardiovascular injury by delivering much less radiation to the heart 28,30. Consistent with prior studies 5,15,19,28–31, we document a lower event risk among women diagnosed with breast cancer in later years compared to women diagnosed in earlier years. We report an effect size that is smaller than previously reported, indicating that modern radiation is associated with a reduced risk of cardiovascular injury. This study also identified a greater risk for the combined outcome in the first 6 months after a breast cancer diagnosis but no statistically significant relationship over time. These findings suggest that women at high risk of cardiovascular events have greater increases in risk from radiotherapy than other women and should be monitored for at least 6 months.

Although prior evidence has suggested no increased risk in cardiac morbidity with modern techniques 28,30, this study has suggested a possible increased risk in events in women with significant medical history of cardiovascular disease. The major finding in this retrospective analysis is consistent with prior studies that have found an increased risk of cardiovascular disease to be associated with the use of radiation in breast cancer 5,20,30. A prior study using SEER-Medicare data found an increased risk for fatal MI after radiotherapy among irradiated women with left-sided breast cancer 20. Similarly, in a prospective study of 10-year survivors treated from 1970 through 1986, the treatment-specific incidence of cardiovascular disease, irrespective of the side of radiation treatment was associated with an increased risk of cardiovascular disease 5. However, our results suggest that the risk of cardiovascular events diminishes over time. Decreases in the risk of major coronary events have been reported in patients with longer time since exposure 32.

While it is true that techniques have evolved to reduce exposure to the heart and the development of treatment planning using computed tomography which enables better visualization of the heart, modern radiation techniques for reducing cardiac exposure have not eliminated the risk of cardiac injury. In the Doyle (2007) study, there was no significant association between radiation and MI (HR = 0.93; 95% CI, 0.84–1.02) or the combined MI and ischemia endpoint (HR = 1.02; 95% CI, 0.94–1.10) that was found. However, this study focused on MI and ischemia as primary endpoints for cardiovascular toxicity. There are other potential adverse effects of radiation exposure to the heart 33 that were not specifically examined, including but not limited to coronary artery disease, pericarditis, cardiomyopathy, valvular disease, heart failure, atherosclerosis, and sudden death.

There are some limitations to this study. It is widely known that radiation-induced cardiac injury has a dose–response relationship 32 where the risk of cardiovascular disease increases with increasing mean cardiac dose; this study did not aim to assess the radiation dose–response relationship. Another limitation is that this study assessed the risk of an event in elderly women treated with any chemotherapy compared to no chemotherapy. Although each chemotherapeutic agent has its unique cardiac effects, this study was not designed to identify which chemotherapy agents are cardiotoxic, but to assess the risk associated with radiation, controlling for potential confounding due to chemotherapy use. A third limitation is that this study could not assess tamoxifen's effects on cardiovascular risk because of the lack of availability of Medicare Part D prescription drug data at the time the study was conducted.

The limitation of available laboratory tests used to diagnose and determine risk of coronary heart disease did not allow the accurate assessment and stratification of women into the high-, intermediate-, and low-risk categories. This is especially true for women in the low-risk group who may have equal chances of being in the intermediate or high-risk group if laboratory data was available. However, the use of ICD-9 diagnosis codes was sufficient to categorize women according to cardiovascular risk groups. The rates of cardiovascular morbidity ascertained are likely to be an underestimate of cardiovascular claims because this study only looked at cardiovascular morbidity that required hospitalization. Other important limitations include potential coding errors in diagnosis and procedure codes that can cause an underestimate or overestimate of true cardiovascular events. The issue of risk is another limitation of this study. This study treated all events in each of the analysis as though they were identical: all deaths were the same and all cardiovascular events were the same. A further limitation of this study and many other clinical studies is that of administrative censoring, which only allowed the observation of events up to the study duration limiting further detection of events.

## Conclusion

This is the first study to assess and stratify women into high-, intermediate-, and low-risk categories to evaluate the risk of a cardiovascular event or death using modern adjuvant radiation for women with differences in cardiovascular risk profiles. Although the use of radiation has made a tremendous difference in treating breast cancer and improving survival demonstrating an overall net survival benefit, however, this study has shown that women with a prior medical history of cardiovascular disease treated with radiation therapy are at increased risk for a cardiovascular event in the first 6 months following diagnosis. Given that the unfavorable effects of radiation is stronger in earlier time periods than in later time periods, women with breast cancer and cardiovascular comorbidity should be monitored by their oncologists and cardiologists for at least 6 months following treatment with radiation therapy.
