# Supplementary material for: Cardiovascular event-free survival after adjuvant radiation therapy in breast cancer patients stratified by cardiovascular risk
Source: Cancer Med. 2014 Jul 10;3(5):1342–52. doi: 10.1002/cam4.283 (PMC4302684; doi:10.1002/cam4.283)
Supplement: Supplementary file 1 — Data S1. Supplementary material. [file cam40003-1342-SD1.docx]

**Supplementary Material**

**Cardiovascular Event:**

**Angina:** 413.0-413.1, 413.9 **Heart Failure:** 428.0-428.1, 428.20-428.23, 428.30-428.33, 428.40-428.43**, Chest Pain:** 786.50-786.51, 786.59, **Ischemia:** 435.9, 414.10-414.11**, Valve Disorder:** 424.0-424.3 **Heart Disease:** 402.00-402.91, 403.00-403.01, **Atherosclerosis:** 414.00 – 414.04, 411.81, 411.89 , 415.11, 415.19**, Conduction/Rhythm Disorder:** 426.X, 427.X, 785.X, 747.0, **Myocardial Infarction:** 410.0 – 410.11, 429.79, **Cardiomyopathy & Elevated Blood Pressure:** 425.0 – 425.1, 425.4, 796.2 **Inflammation:** Myocarditis: 422.90-422.91, 422.93, Pericarditis: 420.90 – 420.91, 420.99

**Radiation**

**CPT Codes:**

77401  77402  77403  77404  77405  77406  77407  77408  77409  77410  77411  77412  77413  77414  77415  77416  77417  77418  77419  77420  77421  77422  77423  77424  77425  77426  77427  77428  77429  77430  77431  77432  77433  77434  77435  77436  77437  77438  77439  77440  77441  77442  77443  77444  77445  77446  77447  77448  77449  77450  77451  77452  77453  77454  77455  77456  77457  77458  77459  77460  77461  77462  77463  77464  77465  77466  77467  77468  77469  77470  77471  77472  77473  77474  77475  77476  77477  77478  77479  77480  77481  77482  77483  77484  77485  77486  77487  77488  77489  77490  77491  77492  77493  77494  77495  77496  77497  77498  77499  77520  77523  77750  77751  77752  77753  77754  77755  77756  77757  77758  7775977760  77761  77762  77763  77764  77765  77766  77767  77768  77769  77770  77771  77772  7777377774  77775  77776  77777  77778  77779  77780  77781  77782  77783  77784  77785  77786  77787  77788  77789  77790  77791  77792  77793  77794  77795  77796  77797  77798  77799  G0256  G0261  77290  77315  76370  76965  36000  36410  77261  77262  77263  77305  77306  77307  77308  77309  77310  77311  77312  77313  77314 77321 77326 77327  77328  77331  77332  77333  77334  77521  77522  77524  77525

**ICD-9-CM Codes:**

922.1 922.2 922.3 922.4 922.5 922.6 922.7 922.8 922.9 923.0 923.2 923.1 923.3 923.9

**Surgery**

**CPT Codes:**

10021 10022 19100 19102 19101 19125 19160 19162 19180 19182 19200 19201 19202 19203 19204 19205  19240 19206 19207 19208 19209 19210 19211 19212 19213 19214 19215 19216 19217 19218 19219 19220 38500 38525 38530  38740  38745 38792 19298 78195 19340 19342 19357 19361 19362 19363 19364 19365 19366 19367 19368 19369 19301 19302  19303 19304  19305  19306 19307 19103 19110 19112 19120 19126 11970 19328 19330 19260 19271 19272 19296 19297

**ICD-9-CM Codes:**

851 852.0 852.1 852.2 852.3 853.3 853.4 853.5 854.1 854.2 854.3 854.4 854.5 854.6 854.7 854.8 402.2  402.3 403 857 858.4 858.5 859.5 855.3 859.4 859.3 85 853.6

**HCPCS CHEMO Codes:**

C1178 J9091 J9070 J9092 J9080 J9090 C9420 C9421 J9093 J9094 J9095 J9096 J9097 J8530  J9130  J9140  C9427  J9208  J9245  J8600  J9320 J9340 J9040 J9120 J9290 J9291 J9280 C9432 J9293 J9270 J8520 J8521 J9065  C9419  J9100  J9110  J9098  J9200  J9185 J9190 J9201 J8610 J9250 J9260 C9213 J9305 J9268  J9150 J9151  J9000  C9415  J9001  J9178  J9180  J9211  J9025  J9206 J9350 J9020 J9266 J8700 J9017 J9600  J9010  C9214  J9035  C9215  J9055  J9300  C1083  J9310  J9355  J9181  J9182  C9425 J8560  Q2017 J9045 J9060 J9062 C9418 C9205 J9263 J9041  J9170  C9431  J9265 J9264  C9127  J9999  J8999  J9360  J9370  J9375 J9380 C9440 J9390

**HCPCS ADMIN Codes:**

C9715 C9714 Q0085 Q0083 Q0084 J9999 C8954 C8955 C8953 C8951 C8950 C8957 C8952 G0921 G0922 G0923  G0924 G0925  G0926 G0927 G0929 G0930 G0931 G0932 96405 96406 96408 96410 96412 96414 96420 96422 96423 96424 96425 96440  96445 96450 96520 96530 96542

**HCPCS NDC Codes:**

000041101 000150503 000150504 000150548 000544129 000544130 000548089 000548130 000153091 003783266 510790965 001730045 595720302 000054507 000544550 003780014 005363998 005550572 599115874 000851244 000851248 000851252 000851259
